# Accurate detection of atrial fibrillation events with R-R intervals from ECG signals

**DOI:** 10.1371/journal.pone.0271596

**Published:** 2022-08-04

**Authors:** Junbo Duan, Qing Wang, Bo Zhang, Chen Liu, Chenrui Li, Lei Wang

**Affiliations:** 1 Key Laboratory of Biomedical Information Engineering of Ministry of Education and Department of Biomedical Engineering, School of Life Science and Technology, Xi’an Jiaotong University, Xi’an, China; 2 School of Electronic Engineering, Xidian University, Xi’an, China; 3 Cardiovascular Medicine, Weinan Central Hospital, Weinan, China; Valahia University of Targoviste: Universitatea Valahia din Targoviste, ROMANIA

## Abstract

Atrial fibrillation (AF) is a typical category of arrhythmia. Clinical diagnosis of AF is based on the detection of abnormal R-R intervals (RRIs) with an electrocardiogram (ECG). Previous studies considered this detection problem as a classification problem and focused on extracting a number of features. In this study we demonstrate that instead of using any specific numerical characteristic as the input feature, the probability density of RRIs from ECG conserves comprehensive statistical information; hence, is a natural and efficient input feature for AF detection. Incorporated with a support vector machine as the classifier, results on the MIT-BIH database indicates that the proposed method is a simple and accurate approach for AF detection in terms of accuracy, sensitivity, and specificity.

## 1 Introduction

Atrial fibrillation (AF or AFIB) is a type of abnormal heart rhythm (arrhythmia) characterized by the rapid, irregular beating of the heart’s upper chambers, resulting in the pooling and clotting of blood inside the heart, thereby increasing the risk of heart attack, failure, and stroke [1]. The symptoms of AF frequently begin with short periods of arrhythmia, such as abnormal beating or atrial flutter, followed by longer arrhythmia periods, sometimes even lasting for hours, accompanied occasionally with heart palpitations, fainting, lightheadedness, shortness of breath, or chest pain [2].

**Fig 1:**
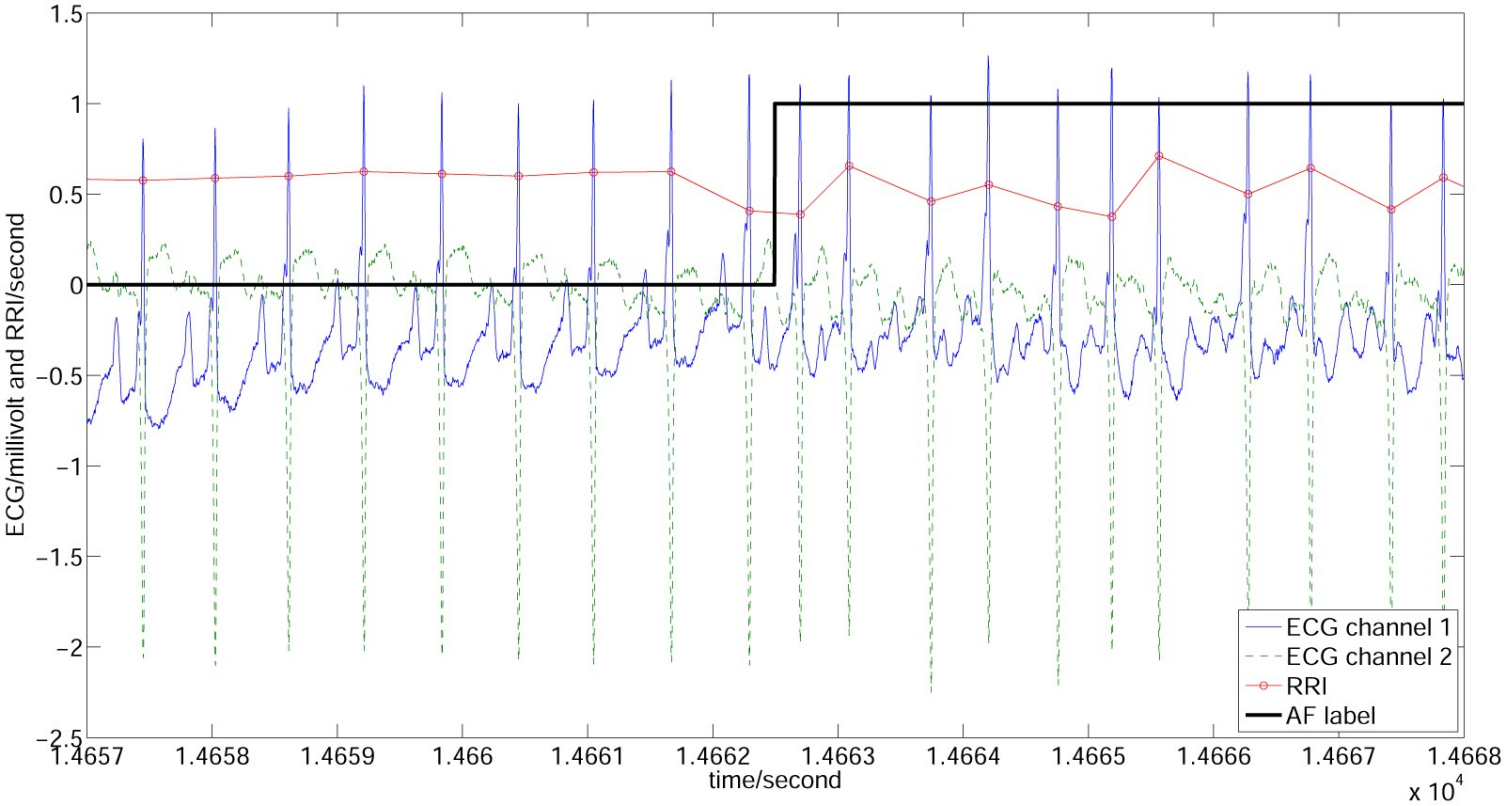
Demonstration of signals and annotations of AFDB (record 04043). Blue solid and green dashed lines indicate the ECG signals of two channels, red circled line is the RRI, and black thick line is the AF label (1 and 0 indicate AF and normal, respectively). It can be easily observed that the RRIs of AF and non-AF segments exhibit different patterns.

The clinical diagnosis of AF is based on the surface electrocardiogram (ECG), and because of the disorganized electrical activity, AF is characterised by the absence of a P wave. However, because the amplitude of the P wave is relatively low (also a heavy baseline), making its detection difficult, the R-R interval (RRI), which reflects the ventricular interbeat, was proposed as a significant biomarker for AF detection [3]. Compared with RRI in regular rhythm segments, consecutive RRIs during AF episodes exhibit low averages and high fluctuations, reflecting rapid and irregular heart beating. Fig 1 illustrates a typical ECG record (04043) from the MIT-BIH atrial fibrillation database (AFDB) [4, 5], which demonstrates the different patterns of RRIs (red line) in and off AF segments. Since AF episodes duration may change from a few seconds to hours, the chance of AF detection depends heavily on the monitoring period of the ECG. To maximize the probability of AF detection, long-term monitoring of the ECG is required, for example, the use of a Holter. However, the visual inspection of twenty-four-hour-long ECG records is time consuming for clinicians. Hence, an automated AF detection method is required. Another issue to be addressed is the distortion in the long-term record introduced by physical activities; therefore, an accurate AF detection method is needed. With the rapid development of wearable and smart devices, memory-efficient, real-time, automatic, and accurate AF detection methods have become possible [6–8].

**Fig 2:**
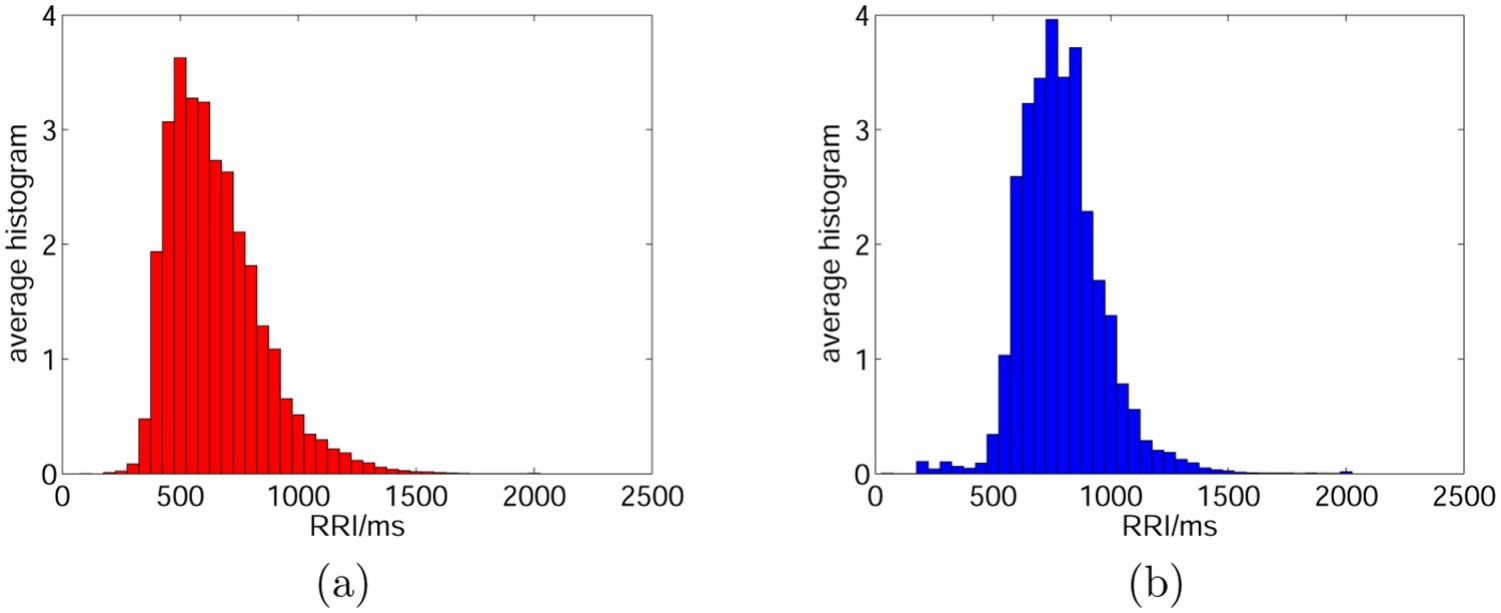
The average histogram of RRIs. (a) represents AF records of AFDB, and (b) represents normal records from NSRDB.

As illustrated in Fig 2, the histogram of the RRI of AF events (panel (a)) exhibits a lower mean, longer tail, and is left-skewed, compared with that of normal events (panel (b)). These findings are evident from Fig 1(b). Based on these findings, mathematical expectation, variance/deviation, skewness, difference of RRIs, and other higher-order statistics were proposed as features for classification.

Table 1 lists 35 published studies that have been conducted to develop efficient AF detection methods. Tateno and Glass [3] first noticed the increase in the variation on AF episodes and hence proposed the coefficient of variation as a feature of RRI and ΔRRI (the first-order difference of RRI). Subsequently, they used statistical hypothesis testing to verify the existence of AF events. They also proposed the use of the Kolmogorov-Smirnov test to compare the histograms of AF RRIs and normal one. Many subsequent studies considered this detection problem as a classification problem and focused on the extraction of various features and the design of classifiers. These features include entropy [9–13], mean and/or median (with or without normalization), root mean square and/or variance [14–16], quantiles [16, 17], median absolute deviation [10, 16, 17], coefficients of wavelet transformation [12, 13], Markov score [18] of RRI and/or ΔRRI, or a combination of several features [10, 11, 16, 19, 20]. In recent studies, deep learning algorithms such as long short-term memory (LSTM) [21, 22], and others [20, 23–25] have been used to process original signals without feature extraction.

**Table 1:** List of published methods (in chronological order).

reference	feature	classifier	database	ACC	SEN	SPE	PRE
[3]	RRI and ΔRRI	K-S test	AFDB,MITDB	NA	86.60	84.30	NA
[14]	RRI(variance)	thresholding	AFDB	NA	96.00	89.00	NA
[26]	RRI and ΔRRI	fixed rule	AFDB	NA	93.00	97.00	NA
[18]	RRI(Markov scores)	thresholding	Holter ECGs,MITDB	95.43	93	98	98.01*
[27]	RRI	thresholding	AFDB,MITDB	99.1	94.4	95.1	106.5*
[28]	RRI and ΔRRI	thresholding	AFDB,NSRDB	NA	96.1	98.1	NA
[9]	RRI(SampEn)	logistic regression	AFDB	97.75	91.00	98.00	63.00
[29]	RRI(map)	thresholding	AFDB,MITDB,NSRDB	NA	95.90	95.40	NA
[19]	RRI(entropys, statistical characteristics), HR	SVM	AFDB,NSRDB,MITDB	98.84	99.07	99.72	98.27
[30]	ΔRRI (maximum), F wave	thresholding	AFDB,MITDB,NSRDB	94.62	94.13	95.58*	97.67
[31]	RRI(ShEn)	thresholding	LTAFDB,AFDB,MITDB,NSRDB	96.05	96.72	95.07	96.61
[32]	P wave (morphology and statistical features)	thresholding	AFDB	97.88	98.09	91.66	79.17
[15]	HR(variance)	SVM	MITDB	97.50	95.81	98.44	97.16*
[33]	RRI(irregularity, Bigeminy suppression)	thresholding	AFDB,NSRDB	NA	98.00	98.20	NA
[34]	TQI(RWE)	NA	AFDB,synthesized ECG recordings	93.32	91.21	94.53	90.53*
[35]	RRI(entropy)	thresholding	AFDB,MITDB	96.38	96.39	96.38	0*
[10]	RRI(CoSEn, CV, RMSSD, MAD)	RF + KNN	MITDB,AFDB,LTAFDB,NSRDB,…	97.33	92.80	98.30	92.10
[36]	RRI(ShEn)	ANN(BP)	AFDB	89.79	91.04	89.01	83.79*
[11]	RRI(ShEn, SampEn, CoSEn, …)	SVM	AFDB	NA	94.27	98.84	NA
[37]	RRI(dissimilarity index)	ensemble classifier	AFDB,NSRDB	97.78	97.04	97.96	92.05*
[38]	ΔRRI(entropy, probability density distribution)	LSVM	AFDB,MITDB,NSRDB,LTAFDB	95.90	95.30	96.30	94.10
[21]	RRI(windowed sequence)	RNN+LSTM	AFDB	98.67	98.51	98.32	100.79*
[12]	ECG(log energy entropy, permutation entropy)	RF	AFDB	96.84	95.80	97.60	96.69*
[16]	HR(statistical characteristics)	fixed rule	AFDB	95.62	95.42	96.12	94.97
[39]	RRI(RCV, SKP, Lempel-Ziv)	SVM	AFDB	96.09	95.81	96.48	97.43*
[22]	RRI(windowed sequence)	CNN+RNN+LSTM	AFDB,MITDB,NSRDB	97.8	98.98	96.95	95.90*
[23]	RRI(sequences)	CNN+RNN+LSTM	private dateset	89.67	94.2	93.13	110.56*
[40]	RRI(entropy, power spectrum …)	SVM	AFDB	90.00	100.00	80.00	83.33*
[41]	RRI(statistical characteristics)	SVCm	AFDB,MITDB	94.99	96.34	92.8	95.6*
[24]	ECG(fractional norm)	H-ELM	AFDB,MITDB	99.93	99.86	100	100.07*
[13]	RRI(frequency-domain)	decision tree	AFDB	98.9	97.93	99.63	98.32*
[42]	HR(ShEn)	thresholding	MITDB	98.10	99.20	97.30	96.39*
[25]	ECG(original wave)	BiRNN	AFDB	82.41	90.53	79.54	61*
[17]	HR(irregularity)	SVM	AFDB	98.66	98.94	98.36	98.86
[20]	ΔRRI, RRI, morphology	CatBoost	AFDB	99.62	99.61	99.64	99.82*

Asterisk (*) indicates that this value is deduced from the other three criteria with formulates in S1 File. Abbreviations: AFDB (atrial fibrillation database), ANN (artificial neural network), BiRNN (bidirectional recurrent neural networks), BP (back propagation), CNN (convolutional neural network), CoSEn (coefficient of sample entropy), CV (coefficient of variance), H-ELM (hierarchical extreme learning machine), HR (heart rate), K-S (Kolmogorov-Smirnov), KNN (k-nearest neighbor), LSTM (long short-term memory), LSVM (linear support vector machine), LTAFDB (long term atrial fibrillation database), MAD (median absolute deviation), MITDB (MIT-BIH arrhythmia database), NA (not applicable), NSRDB (normal sinus rhythm database), RCV (robust coefficient of variation), RF (random forest), RMSSD (root mean square of the successive differences), RNN (recurrent neural network), RRI (R-R interval), SampEn (sample entropy), ShEn (Shannon entropy), SKP (skewness parameter), SVCm (supervised contractive map), SVM (support vector machine), TQI (T-Q interval).

In this study, from a statistical perspective, we consider that instead of employing any numerical characteristic (*i.e*., mean, variance, skewness, *etc*.) as a specific feature, the probability density function conserves comprehensive information and hence enables high-performance classification. Consequently, we propose the use of a histogram of the RRI from an ECG as a natural and general feature and the widely used support vector machine (SVM) as the classifier.

## 2 Materials and methods

### 2.1 Databases

This study employed the MIT-BIH atrial fibrillation database (AFDB) [4, 5], which is widely used in arrhythmia studies. This database includes 25 records of human subjects with AF, and each record includes two-channel ECG signals with a sample frequency of 250 Hz and a 12-bit A/D resolution. Furthermore, this database contains clinical annotations and QRS calls, and supports online retrieval with the easy-to-use toolbox waveform database (WFDB) [43, 44]. Note that R waves were already called by WFDB, so this paper do not cover the detection of R waves from an ECG signal. Researchers interested in this topic are referred to fruitful literature [45–48].

Fig 1 illustrates a typical record (ID 04043) of the AFDB, including two ECG channels (blue and green lines), the RRI (red line), and AF label (black line).

The MIT-BIH long-term atrial fibrillation database (LTAFDB) [49, 50] was also employed as a positive test dataset, which includes 84 long-term (24 hours) ECG recordings, with the same sampling parameters as the AFDB.

As in normal control cases, to evaluate the specificity, this study also employed the MIT-BIH normal sinus rhythm database (NSRDB) [51, 52], which includes long-term ECG records of 18 human subjects who exhibited no significant signs of arrhythmia.

All 127 (25 + 18 + 84) records were downloaded using MATLAB (the pseudo code is listed in S2 File).

### 2.2 Performance criteria

Three widely used criteria were employed to quantify AF detection performance: accuracy (ACC), sensitivity (SEN), and specificity (SPE).

SEN is referred to as the true positive rate, which is used to measure how well a method can identify real patients, and is defined as the proportion of true positives among all positive subjects.

SPE is referred to as the true negative rate, which is used to measure how well a method can identify a normal person and is defined as the proportion of true negatives among all negative subjects.

For diagnosis and screening, there exists a trade-off between SEN and SPE; therefore, ACC is commonly used to consider SEN and SPE integrally. ACC is defined as the proportion of the sum of true positives and true negatives among all the samples.

Notably, precision (PRE), also known as positive predictive value (PPV), is frequently employed in several studies and is defined as the proportion of true positives among all detected positive cases. However, among these four criteria (ACC, SEN, SPE, and PRE) only three are independent, and the fourth can be calculated depending on the other three (see S1 File). Therefore in our study, only ACC, SEN, and SPE were evaluated, and PRE was not considered. Among the studies listed in Table 1, a few studies provided PRE, while others provided ACC; hence, we used the formulas mentioned in S1 File to convert among them, and the resultant values have been labelled with asterisks.

### 2.3 Method

#### 2.3.1 Data pre-processing

After 127 records were downloaded, the following pre-processing steps were followed:

1. RRI values were re-scaled from the sample index to milliseconds by dividing with the sampling frequency;
2. Annotations and comments of AFDB and LTAFDB were resolved, and RRI regions with the string ‘(AFIB’ were selected as positive regions;
3. All RRI regions of NSRDB were selected as negative regions;
4. Both positive and negative regions were cut to segments, each including 30 PPIs;
5. A histogram with *M* bins of each RRI segment was calculated, and stored in a row vector of size *M*;
6. The *N*_0_ row vectors from NSRDB were cascaded vertically to form the negative sample matrix ***X***_0_, and the same method was employed for vectors from AFDB and LTAFDB, yielding matrices ***X***_1_ and ***X***_2_ of height *N*_1_ and *N*_2_, respectively.

After pre-processing, we obtained three sample matrices ***X***_0_, ***X***_1_, and ***X***_2_, with width *M*, and height *N*_0_ = 58742, *N*_1_ = 16817, and *N*_2_ = 101376, respectively.

#### 2.3.2 Classifier

Soft-margin support vector machine (SVM) [53, 54] was trained as the classifier, which is formally defined as the following optimization problem (the Lagrangian dual form):

$$\begin{array}{ccc} & & \underset{\alpha }{\text{max}}\{\text{sum}\left(\alpha \right)-\frac{1}{2}{\left(\alpha ⊙y\right)}^{T}K_{X}\left(\alpha ⊙y\right)\},\\ & & s.t.\;\alpha^{T}y=0,\;0⪯\alpha ⪯c, \end{array}$$ (1)

where $X=[x_{1};x_{2};\ldots ;x_{N}]\in R^{N\times M}$ stores the *N* training samples, each sample $x_{i}\in R^{1\times M}$ is a row vector of length *M*; $y\in R^{N\times 1}$ stores the labels of samples (1 for AF, and -1 for normal); $\alpha \in R^{N\times 1}$ is an unknown weight vector to be optimized; sum(***α***) is the sum of all elements in ***α***; ⊙ is the point-wise multiplication (the Khatri-Rao product); *c* is a box constraint parameter, which controls the strength of regularization; $K_{X}=[k_{ij}]\in R^{N\times N}$ is the kernel matrix of ***X***, with element *k*_*ij*_ = *κ*(***x***_*i*_, ***x***_*j*_) is the Gaussian kernel (or radial basis function):

$$\begin{array}{c} \kappa \left(x_{i},x_{j}\right)=\text{exp}\;(-\frac{\|x_{i}-x_{j}{\|}_{2}^{2}}{\sigma^{2}}), \end{array}$$ (2)

where *σ* is a scale parameter.

To optimize problem (1), sequential minimal optimization (SMO) [55] was utilized as the solver. Once ***α*** is obtained, the bias parameter *b* can be calculated as:

$$\begin{array}{c} b=\text{mean}(y-K_{X}\left(\alpha ⊙y\right)). \end{array}$$ (3)

For a test PPI vector ***t***, the predicting function read:

$$\begin{array}{c} p\left(t\right)=\sum_{i=1}^{N}\alpha_{i}\kappa \left(x_{i},t\right)+b, \end{array}$$ (4)

and if *p*(***t***) > 0, an AF event is detected.

## 3 Results

### 3.1 Kernel function

First, we compared the performance of the Gaussian kernel function (2) with that of the linear kernel function $\kappa_{l}\left(x_{i},x_{j}\right)=x_{i}x_{j}^{T}/\sigma^{2}$, and the third-order polynomial kernel function $\kappa_{p}\left(x_{i},x_{j}\right)={\left(1+x_{i}x_{j}^{T}/\sigma^{2}\right)}^{3}$. At this step, all other parameters were set to default values (scale parameter *σ* = 1, box constraint parameter *c* = 1, and number of bins *M* = 10).

The SVM classifier was trained with the positive and negative sample matrices ***X***_1_ and ***X***_0_ by constructing a kernel matrix ***K***_*X*_ of size (*N*_1_ + *N*_0_) × (*N*_1_ + *N*_0_), and a training label *y* with *N*_1_ ones and *N*_0_ negative ones. The optimize problem (1) was solved with SMO solver to train the weight vector *α*; then, the bias parameter *b* was calculated based on Eq (3). Subsequently, the same samples were tested with the trained SVM, and the performance criteria were evaluated.

**Fig 3:**
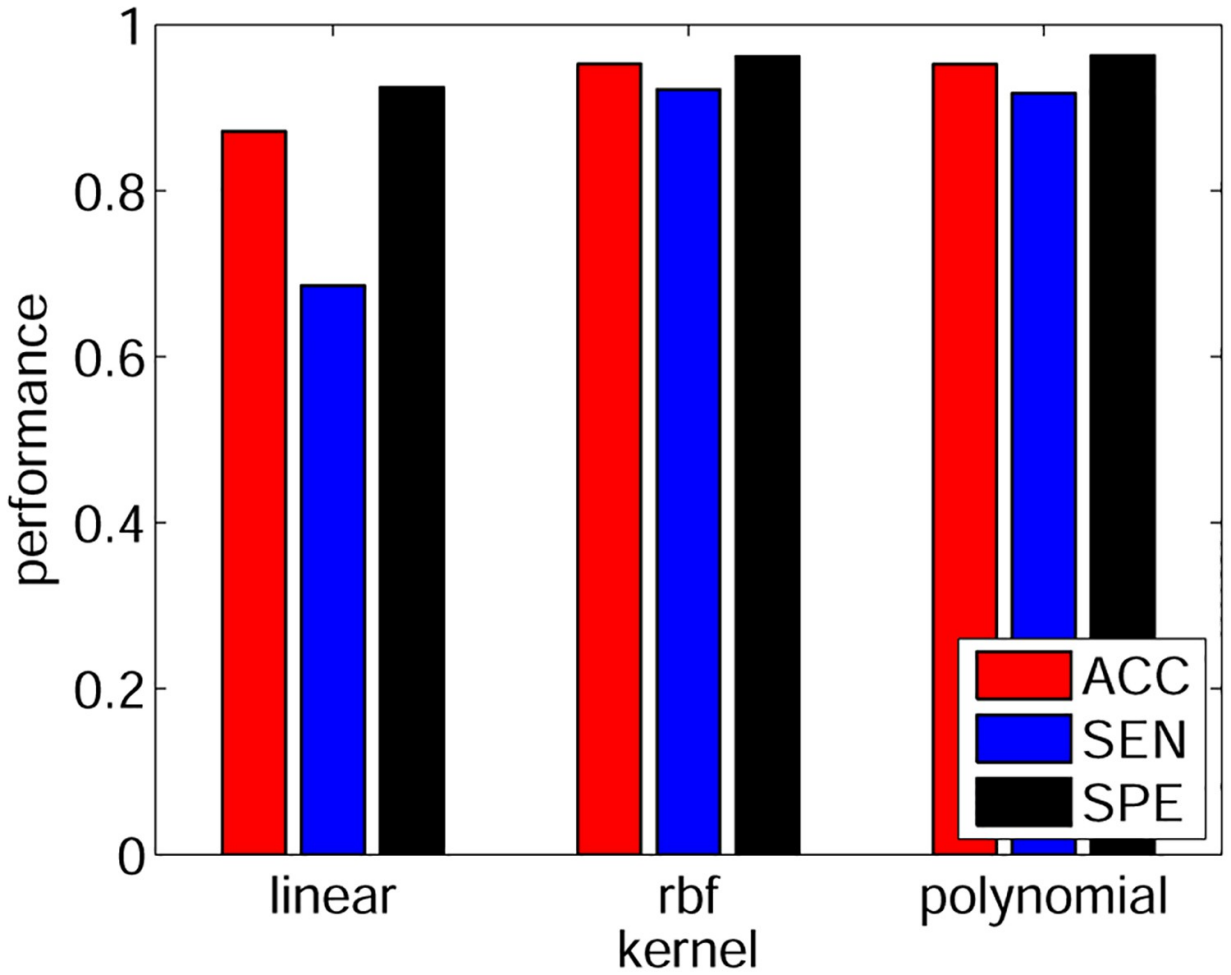
The performance with different kernel functions.

Fig 3 illustrates the performance with different kernel functions. It was demonstrated that the radial basis function was the best, and this kernel was chosen in the sequel.

### 3.2 Number of histogram bins

Subsequently, we tested the impact of the histogram bin number *M* on detection performance. Because approximately 99% of the RRI values lie within the region of 50 ms and 2e3 ms, the centres of the first and last bins were set to 50 ms and 2e3 ms, respectively. Other *M* − 2 bin centres were located linearly within this region. RRI values beyond this region were assigned to either the first or the last bin.

**Fig 4:**
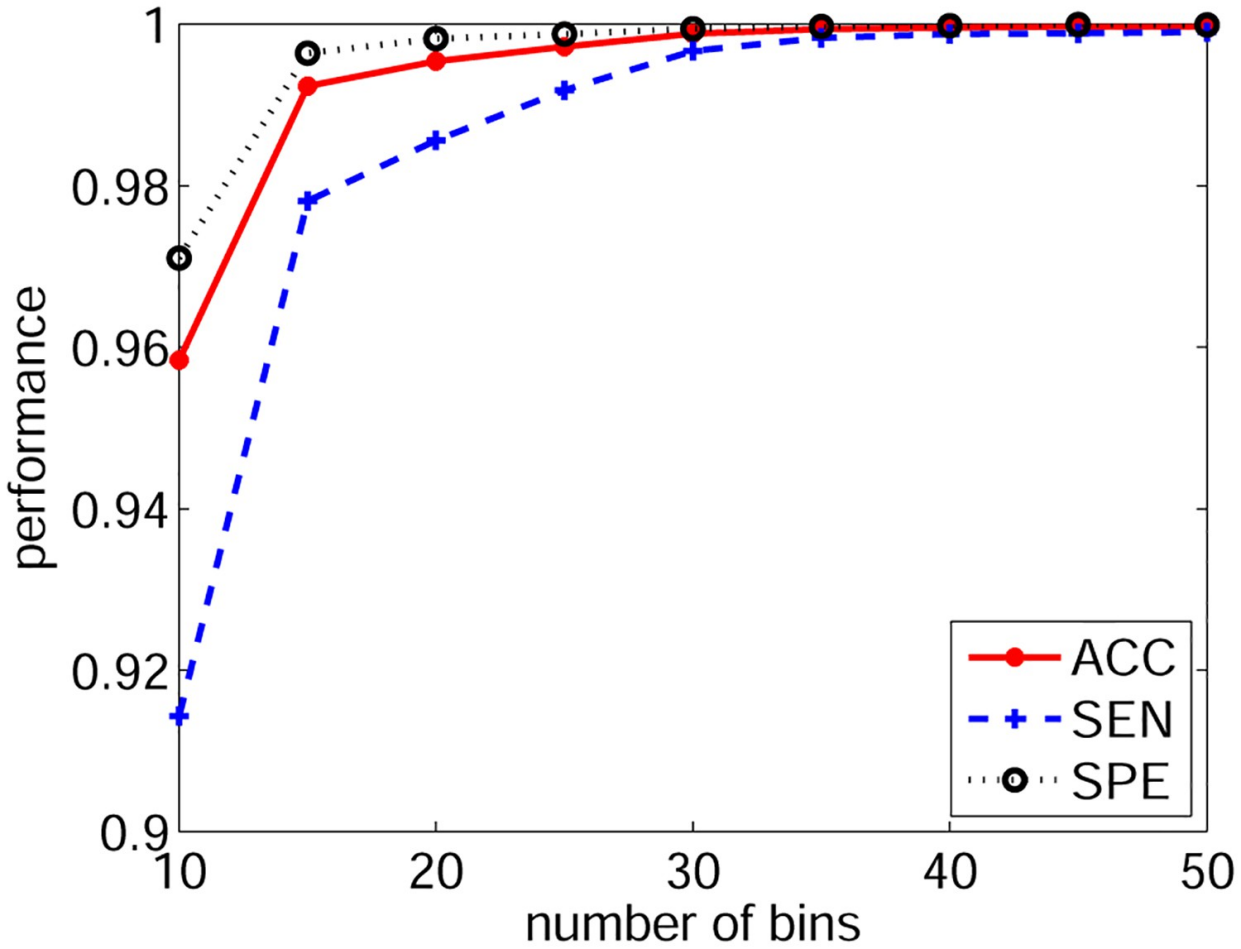
The performance with different histogram bin numbers.

The training of SVM and performance evaluation were the same as in the previous experiment. Fig 4 demonstrates the results, which indicate that the detection performance increases with an increase in *M* and reaches the ceiling at 30. Therefore, in the following experiments, *M* was fixed at 30.

### 3.3 Cross-validation with scale and box constraint parameters

**Fig 5:**
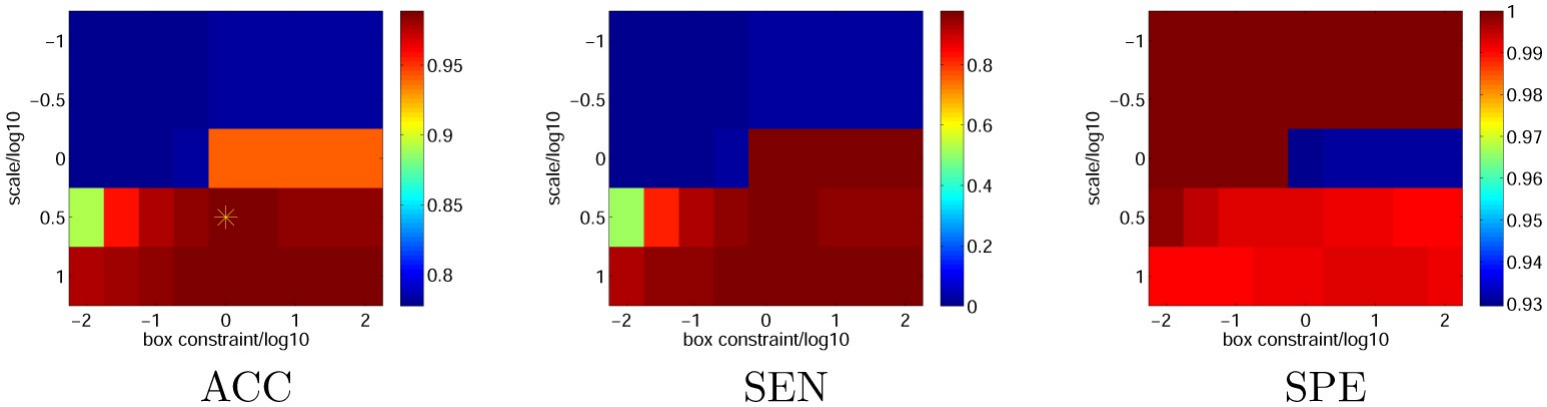
Ten-fold cross validation performance with different scale and box constraint parameters.

The scale parameter *σ* and box constraint parameter impacted the training significantly; hence, we used ten-fold cross-validation to optimize these two parameters. Both *σ* and *c* were sampled on a two-dimensional logarithmic grid. The training of SVM, performance evaluation, and training dateset were the same as in the previous experiment. Fig 5 demonstrates the results in which panel (b) indicates that a high SEN performance requires large-scale and box constraint parameters, and panel (c) indicates that a high SPE performance requires small-scale and box constraint parameters. As mentioned, ACC considers both SEN and SPE, and the best performance was achieved at *σ* = 3.2, and *c* = 1 (the yellow star in panel (a)). Table 2 lists the ten-fold cross-validation performance in this setting.

**Table 2:** Performance of ten-fold cross validation at yellow star point in Fig 5.

CV ID	ACC	SEN	SPE
1	0.9876	0.9878	0.9875
2	0.9839	0.9862	0.9823
3	0.9859	0.9896	0.9832
4	0.9849	0.9812	0.9878
5	0.9847	0.9865	0.9833
6	0.9837	0.9853	0.9826
7	0.9839	0.9828	0.9848
8	0.9854	0.9852	0.9856
9	0.9827	0.9836	0.9821
10	0.9805	0.9801	0.9808
average	0.9843±0.0019	0.9848±0.0029	0.9840±0.0024

### 3.4 Independent dataset testing

In the last experiment, the SVM model was trained with AFDB (*N*_1_ = 16817) and NSRDB (*N*_0_ = 58742) as positive and negative samples, respectively. Model parameters were set according to the results of previous experiments. Subsequently, LTAFDB (*N*_2_ = 101376) was used as the independent positive testing dataset. The confusion matrix is shown in Table 3, and detection results for ACC, SEN, and SPE were 0.9697, 0.9524, and 0.9994, respectively, thus indicating a good generalization performance.

**Table 3:** Confusion matrix of independent dataset testing.

	predicted AF	predicted normal
AF	96553	4823
normal	34	58708

## 4 Conclusion and discussion

We conclude that an accurate detection method for atrial fibrillation events based on the RR interval measured from an ECG signal was proposed in this paper. The advantage of the proposed method over the methods described in literature is that: instead of using any specific numerical characteristic (*e.g*., entropy, mean, median, root mean square, variance, quantiles, *etc*. or a combination of several characteristics) as the input feature, the probability density conserves all statistical information; hence, is natural, comprehensive, easy-computing and efficient as the input features. On the MIT-BIH databases, the proposed method achieved 0.9843±0.0019, 0.9848±0.0029, and 0.9840±0.0024, in terms of ACC, SEN, and SPE, respectively, for a ten-fold cross-validation, and 0.9697, 0.9524, and 0.9994, respectively, for an independent testing, indicating that the proposed method is effective in AF detection.

Note that some studies highlighted the difference between the histograms of PPIs of AF and normal one, and proposed the use of a histogram to detect AF, but the manner in which they utilize histograms is quite different from that in this study. For example, Tateno and Glass [3] observed an increase in variation in AF episodes, and proposed using the Kolmogorov-Smirnov test to compare the histogram of AF RRI and normal RRI; Petrucci *et al*. [26] calculated several statistics, such as the distribution width based on the histogram of RRI prematurity and ΔRRI, and used a geometric test to detect AF. Alternatively, this study proposes using the histogram as the feature vector and an input to the support vector machine for classification, which is the main contribution of this study.

## Supporting information

S1 File Relations between accuracy (ACC), sensitivity (SEN), specificity (SPE), and precision (PRE). (PDF)

S2 File Dataset downloading. (PDF)
