# Supplementary material for: Accurate detection of atrial fibrillation events with R-R intervals from ECG signals
Source: PLoS One. 2022 Aug 4;17(8):e0271596. doi: 10.1371/journal.pone.0271596 (PMC9352004; doi:10.1371/journal.pone.0271596)
Supplement: S1 File — (PDF) [file pone.0271596.s001.pdf]

Supporting information of "Accurate detection of atrial fibrillation events with R-R intervals from ECG signals"

S1 FILE: RELATIONS BETWEEN ACCURACY (ACC), SENSITIVITY (SEN), SPECIFICITY (SPE), AND PRECISION (PRE)

$$ACC = \frac{SEN((1-2PRE)SPE + PRE)}{(1-PRE)SEN + PRE(1-SPE)} \tag{1}$$

$$SEN = \frac{ACC(SPE-1)}{ACC(1/PRE-1) + SPE(2-1/PRE) - 1} \tag{2}$$

$$SPE = \frac{1 + ACC(1-1/PRE-1/SEN)}{2 - ACC/SEN - 1/PRE} \tag{3}$$

$$PRE = \frac{SEN(ACC-SPE)}{ACC(SEN+SPE-1) + SEN(1-2SPE)} \tag{4}$$
