# Supplementary material for: Accurate detection of atrial fibrillation events with R-R intervals from ECG signals
Source: PLoS One. 2022 Aug 4;17(8):e0271596. doi: 10.1371/journal.pone.0271596 (PMC9352004; doi:10.1371/journal.pone.0271596)
Supplement: S2 File — (PDF) [file pone.0271596.s002.pdf]

Supporting information of "Accurate detection of atrial fibrillation events with R-R intervals from ECG signals"

S2 FILE: DATASET DOWNLOADING

The pseudo MATLAB code of dataset downloading is listed in the following table:

TABLE I
PSEUDO DOWNLOAD CODE OF AFDB, LTAF, AND NSRDB WITH WFDB MATLAB TOOLBOX.

```
for i=1:length(list_file_name)
    [signal{i},frequency_sampling(i),time_signal{i}]=rdsamp(list_file_name(i));
    [annotation{i},type{i},subtype{i},channel{i},NUM{i},comments{i}]=
            rdann(list_file_name(i),'atr');
    gqrs(list_file_name(i));
    [RR{i},time_RR{i}]=ann2rr(list_file_name(i),'qrs');
end
```

The following functions from the WFDB toolbox were used: *rdsamp ,ann2rr, gqrs, rdann*, where *rdsamp* reads the ECG signals and sampling information in the specified record *list_file_name(i)*, *rdann* reads the annotation files, *gqrs* generates the QRS complexes in an ECG signal, and *ann2rr* converts the annotation files to generate the RRI.
